# Chief Nursing Officers’ Views on Meeting the Needs of the Professional Nurse: How This Can Affect Patient Outcomes

**DOI:** 10.3390/healthcare6020056

**Published:** 2018-05-31

**Authors:** Charlene Ingwell-Spolan

**Affiliations:** School of Nursing, University of Maine, Orono, ME 04469, USA; Charlene.ingwellspolan@maine.edu; Tel.: +1-207-581-2605

**Keywords:** leadership, nursing leadership, patient outcomes, healthy work environment, nurse education

## Abstract

Chief Nursing Officers (CNOs) have a demanding, complex role that commands accountability in leading the nursing profession and achieving quality patient outcomes. The purpose of this study was to understand the CNO’s view of meeting the needs of the Registered Nurse (RN) at point of care and how this could affect quality patient outcomes. In two qualitative studies twenty-five CNOs were individually interviewed in eight states including: Florida, Tennessee, Kentucky, Maine, New Hampshire, Vermont, Massachusetts, and New Jersey. The majority of these CNOs interviewed believed they were doing the best for their nurses and their healthcare facility. After analyzing their responses, it was apparent that some CNOs actually encouraged peer pressure among nurses to achieve compliance and felt patient acuity is being addressed adequately, since most patients were discharged within three to four days and those that were more critical were admitted to the critical care units. The average length of stay, which is the number of paid days a patient remained in the hospital, was an important metric. A large amount of nurses felt they were unable to deliver the care needed for their patients due to patient load, lack of collaboration among the health care team, higher patient acuity and absence of decision-making and autonomy. Many of the CNOs trusted that patient care outcomes, meaning relatively short hospital stays, demonstrated that the nursing practice was successful; rather than first having the nurse being set up for success to provide the best care possible to their patients.

## 1. Introduction

The demanding, complex role of Chief Nursing Officers (CNOs) today commands accountability in leading the professional nurse to help achieve consistent quality patient outcomes. Twenty-five CNOs were interviewed individually in two qualitative research studies from eight states including: Florida, Tennessee, Kentucky, Maine, New Hampshire, Vermont, Massachusetts, and New Jersey. These phenomenological inquiries examined the CNOs’ perspectives of meeting the needs of the Registered Nurse (RN) at point of care and how they felt this affected quality patient outcomes.

Today’s RNs’ experiences often include: job dissatisfaction; high patient acuity; lack of autonomy and decision making; plus bullying in the workplace [1,2,3,4]. These barriers in the acute care setting result in a less supportive work environment and may prevent the RN from delivering quality patient care [5,6,7,8,9]. The CNO is the leader of the professional nurse and is ultimately responsible for the actions and interventions of the RN at point of care [10]. Metrics are in place to measure patient care outcomes; e.g., The Hospital Consumer Assessment of Healthcare Providers and Systems (HCAHPS), physician/health care colleagues’ reactions/complaints, and patient incident reports. There is intense pressure for the CNO to maintain high scores on these measures; all required for the ultimate success of the CNO [9,11,12,13].

## 2. Background

In prior studies, RNs describe their jobs as stressful [2,7]. Negative perceptions of the work environment can be predictors of the desire to leave nursing [2,7] and can also be a cause for an even lower commitment to the institution and profession by newly licensed RNs, affecting nurse retention, overall attrition rates, and patient outcomes [1,6]. Likewise, these additional factors negatively affect the success and length of CNO employment [14]. 

Nurse-assessed quality of care and better RN job satisfaction at point of care [9,12] are influenced by strong leadership, organizational culture, and group cohesiveness [9,12]. A cross sectional survey [6] showed that there is a relationship between a transformational leadership style and quality of care; facilitated through organizational and peer support, autonomy, and workload of the RN.

A mixed method study [11] showed a linkage between organizational climate and nurses’ performance of caring practices in hospitals showing consistency with the Quality Caring Model [15,16]. This model promotes caring with relationship building concepts that nurses demonstrate within their daily clinical practice. These relationship centered encounters are with patients, families, and the health care team and are related to improved patient care outcomes [16]. The workload of the RN is the most obvious factor influencing caring practices and patient outcomes [11,15,16]. Leadership and teamwork linked to role clarity for nurses and characteristics of patients/families such as relationships, support systems, and past history have distinct influences on RNs caring and quality patient outcomes [11,15,16].

The CNO is the leader of the RN at point of care. Remarkable leaders keep themselves informed on what is going on in the workplace, welcoming ideas and opinions that are intended for the improvement of performance. They also see the significance in knowing the truth within the current experiences of the daily work environment. Finding the truth and making people feel comfortable and safe in communicating relevant information are attributes of successful and renowned leaders [17]. 

The CNO needs to move beyond dependence on current clinical models in which the RN at point of care implements physicians’ orders, is a subordinate member of the health care team and is not comfortable in voicing their observations in regards to their patients’ care. Rather, professional nurses, under the direction of the CNO need to become more skilled and interdisciplinary team members. This requires high-level communication and advocacy for nurses to become equal partners in health care. Speaking up about organizational issues and suggesting changes to operating procedures may cause risk to the person speaking; however, when spoken effectively this could be viewed as constructive [18,19]. Research has found that CNOs do not have consistent, standardized leadership education that facilitates success in these areas regarding the RN at point of care in the healthcare industry [9,10,17].

Professional barriers in the workplace, fragmentation of clinical systems, and administrative disconnect can prevent the professional nurse from articulating new models for patient care. Many times nurses are systematically excluded from contributing information crucial to patient decision-making and quality of care [6,8,18]. Nurses must find a way, institutionally, to overcome the barriers that prevent them from enacting their professional model of care. This will involve nursing staff communicating and negotiating interpersonally, socially, and organizationally, as well as clinically; which are imperative to further the trust of patients, families, and the wider population [18,19]. 

For example; a platform for change at patient point of care focusing on the CNO and Chief Financial Officer’s (CFO) relationship in addressing challenges of quality patient care, safety, and financial performance found that CNOs communicating with CFOs and Chief Executive Officers (CEO) involves understanding financial constraints and balancing them with expected quality patient outcomes [13]. This financial knowledge from the nursing perspective should be communicated by the CNO to the CFO as well as to the professional nurses. With ever-increasing pressure to cut costs the partnership between nursing and finance needs to tackle these new challenges. This collaboration has historically been strained and does not always come easily due to differences in focus, priorities, and disparate communication. In this case, effective communication between the CNO and CFO resulted in the development of benchmarks, a functional nurse staff council, an evaluation process of patient acuity, and classification systems to prevent inequitable assignments [13]. These sorts of proven effective models of communication and collaboration are imperative not only for the success of the CNO but for the RN at point of care and most importantly for quality patient outcomes.

Therefore, from the literature it is known that a healthy work environment including effective communication, advocacy, and interdisciplinary cooperation facilitates consistent quality patient outcomes [20,21]. Since the CNO is the highest nursing decision maker, communicator, and collaborator she/he is best able to set the required standards to achieve a healthy work environment for nursing that provides the catalyst for consistent quality patient outcomes. 

During the literature review there were no qualitative studies of CNOs’ perceived work experiences in the acute care setting. As a result two qualitative studies, an initial study and a replication study, were completed and combined by this author in which CNOs voiced their views on meeting the needs of the professional nurse and its effect on patient care outcomes. 

## 3. Materials and Methods

The purpose of this author’s initial and replicated study was to explore the lived experience of the CNO as the lead voice for the professional nurse. The substance of the interviews consisted of “what” they experienced and “how” they experienced it. Following the expansive, overarching research questions were the follow-up interview questions and prompts in which this portion of both studies explored the CNOs’ view on meeting the needs of the RN at point of care and how they felt this affected quality patient outcomes. The researcher’s own prejudgments were set aside through epoch. Trustworthiness is equated to validity in quantitative research [22]. This was implemented through one-on-one interviews and member checks which provided the participants an opportunity to give feedback on the identified themes. Trustworthiness is not established as it is created and cultivated. This is accomplished through methodical collection of data and in-depth analysis of the data; yielding, thick, rich descriptions of the phenomenon of studies that has been clearly saturated. The continuous bracketing of the researcher served as evidence of the researcher’s honesty. Dependability was achieved as each participant reviewed his or her verbatim transcript along with confirmability as the results were confirmed or collaborated by others showing details of the methodology used. 

CNO participants were accessed through professional nursing leadership organizations and through purchased targeted contact lists. Email invitations were sent to future participants advising them of the general scope of the studies.

Institutional Review Board (IRB) approval was received for both the initial study and the replicated study. Inclusion criteria for the two studies included: CNOs within an acute setting, a hospital which has facilities and all personnel including medical staff appropriate to diagnose, treat and care for acute conditions, including injuries, located within the Southeastern and Northeastern United States. CNOs were identified as the highest ranking administrative registered nurse in the acute care organization, responsible for the practice of nursing throughout their healthcare system. Exclusion criteria included anyone not meeting the inclusion criteria. Department directors, division directors, unit or service managers, supervisors, charge nurses and other senior nurses who have non-nursing, executive positions in hospitals were also excluded.

Both studies were explained to the participants prior to the interviews, in which all questions were answered in regards to the informed consent. All the participants who met the inclusion criteria and who agreed to participate in the study then signed the informed consent. Twenty-five CNOs were interviewed using Moustakas’ transcendental phenomenological inquiry approach, determining the overall essence of the CNOs’ lived experience as the lead voice for the RN in the acute care setting. 

The two research questions viewed appropriate for this portion of the two studies in accordance with Moustakas’ design were: (1) How do you meet the needs of the RN at point of care? and (2) Does meeting the needs of the RN affect patient care outcomes?

A naturalistic inquiry research approach was integrated through the direct experience of the CNOs. This model-included context, perspectives, experiences, underlying motivations and factors that influence decision making and opinions of CNOs. 

The studies included six male and nineteen female CNOs actively practicing in Florida, Tennessee, Kentucky, Maine, New Hampshire, Massachusetts, Vermont, and New Jersey. The participants’ ages ranged from early forties to late sixties. They were educated to at least a master’s level up to a PhD degree and were primarily Caucasian. The CNOs in these studies generally held several CNO positions in the last decade. The types of hospitals represented included; corporate system, community, government, academic health center and rural facilities. The number of beds per hospital ranged from less than 50 to over 1000.

In accordance with Moustakas’ Transcendental Phenomenology, there were multiple reviews of transcriptions following each interview with member checks. These reviews led to identification through horizontalization and delimited meanings of the invariant qualities in which patterns and themes emerged. From these patterns and themes, an integrated textural and structural description was completed per participant. 

## 4. Results

After careful and studied consideration and analysis of the phenomenon being studied, two essential themes emerged in regards to meeting the needs of the RN at point of care and how this affects quality patient care outcomes. These two themes were advocating and conflicting. The CNOs were continuously advocating for the patient and many times this advocacy for the patient could be conflicting with the needs of the nurse. Following are examples of the transcriptions obtained in these two studies.

The Chief Nursing Officer, by virtue of the title, might be assumed to chiefly represent nursing. However, all CNOs interviewed in this study noted their primary goal and responsibility was to the patient. According to these CNOs, this may or may not coincide with the best interest and objectives of the nurses. This is the conflict that is one of the primary themes that emerged from these two studies.

As one CNO described this conflict:

“People know that I have the patient’s best interests at heart. Because I have to think what’s best for the patient, not best for the nurses. What’s best for the patients right now might be what’s best for the nurse’s today. But it might not be…”

Another CNO explained their role as an advocate for the patient even though they are the Chief Nursing Officer:

“That is why I round on patients, because that reminds me why I do what I do every day. … because I am a nurse first … I’m doing it for the patient in the bed … because my priority is the patient’s safety.”

Even though patient care and patient care outcomes are the primary goal of the CNOs interviewed, many were apprehensive and some even cautioned by their executive teams to not foster dissension among nurses which might result in outcomes detrimental to the organization including; nurse turnover, loss of reputation, and union activity. This at times caused the CNO to feel frustrated in bringing new ideas and processes to the workplace for concern this would cause discord among the RNs. 

As one CNO stated this conflicting dilemma:

“No one wants the nurses trying to bring in a union … picketing in front of the hospital. So, you got to keep the nurses happy … but there is also this extreme pressure to get the largest workforce in the hospital to perform well for everyone else.”

In order to be an effective leader, the individuals you lead must have a work environment conducive to success, feel valued, have opportunities to advance professionally and have a voice. Only one of the CNOs interviewed seemed less conflictive and was an advocate for the nurse as they identified this connection; that if the nurses are allowed to self-actualize, the patient will be the ultimate beneficiary. The CNO stated: “If my nurses are well taken care of, my patients will be taken care of.”

Many of the CNOs talked about having better work processes, documentation systems, and call systems for the RN at point of care. In practice, evidence of meeting goals and objectives of the non-nursing leadership team took priority. For example, peer pressure among nurses was utilized to meet staffing needs. Another example of a CNO’s conflict between believing they are an advocate for the professional nurse is that they were actually influencing nurses to work overtime by encouraging nurses to coerce other nurses to do so:

“We never pressure for overtime, it’s just not a good work environment … you do a neutral approach and people contribute if they’re part of a team … there are always some that do more than others … if you have somebody who never does it that brings the team down … you address it more from a team performance … after a while the peers will look and resent it … If they see the team doing it everybody pitches in…”

Many of the CNOs were concerned about length of patient stay, since this affects revenue. However, they were generally not concerned if, e.g., nursing assessments were not being done since they are not evaluated on this standard. Yet, assessments are being documented as always being completed, even though they are not always fully or even substantially completed. As one CNO noted, patient assessments were not important, however, RN’s at point of care feel they do not have the ability to give best patient care such as assessments. They also feel they do not have adequate time to spend with each patient. This CNO expressed continuous conflict; such as revenue versus patient care and advocating for nurses without providing appropriate time for assessment and other interventions at point of care:

“I don’t think (assessments) are very thorough … I mean the length of stay is 4 days. The median is probably 3.1. I don’t know how important it is, you know? I will tell you that there are a lot of things I would like to improve in terms of having nurses spend more time at the bedside … if they had more time, they would probably do more things … for patients … but within a few days … they go home.”

When asked about patient acuity and if this is factored into the success of the RN, one CNO stated using the term “nursing acuity” that this may not be an issue as nurses do have enough time for even the most complex patient interventions. However, most RNs at point of care feel they do not have the time to give the best care to their patients which contributes to an unhealthy work environment. This CNO did not perceive the conflict, instead felt that nurses are adequately advocated for, stating:

“When you think about nursing acuity systems, even if patient load … is about 20 percent more than what you would expect … on the patient load of four patients … what are you going to need, eight and a half hours on your shift? … So I am not believer that we’re that nuanced … Teaching and talking to them, talking to the family, I mean, that’s about it. Even the most complex dressing change doesn’t take very long … the nursing things are 1000 one minute things.”

Another CNO discussed that patient care outcomes revolve around having enough resources for the nurses. This CNO explained how advocating for nurses helped achieve good results for the patient:

“I got great outcomes because we didn’t have a lot of resources, … because I was in a position as a COO, I ratcheted down all the non-nursing departments and ran them lean as anything, but ran nursing at a higher level of nursing hours per patient per day. And that did give great outcomes for the hospital … when nursing has a voice at the table at the board level and at the senior executive level, you can do good things. The key is getting well-trained people … however, if you have folks like here, that … are getting what they’re getting because they’re compromising and, you know, Ms. Nicey-nice and stuff … that can be to the detriment of nursing … they don’t want to take the risks and fight the fight. And you’ve—got to—you’ve got to be a fighter to be a—a successful nurse executive. You can’t sit back…”

## 5. Discussion

These two studies found are groundbreaking in the area of qualitative research on the lived experiences of CNOs. There were no comparable studies prior to these.

The majority of CNOs interviewed for these two studies were considered highly clinically competent early in their careers. The skills required later at the executive level were not the focus of their undergraduate and graduate level of education. A two‑year initiative by the Robert Wood Johnson Foundation (RWJF) and the Institute of Medicine (IOM) found that the undergraduate and graduate education of nurses in the academic setting does not address the realities of health care [23]. The need for highly educated nurses to manage complex healthcare systems, build relationships with healthcare teams in order to collaborate and coordinate across all specialties and professions within the healthcare industry is paramount to achieve better patient outcomes. This will be accomplished by reinventing the nursing curriculum to include all aspects of competency in leadership, health policy, systems, research, and evidence based practice [23]. In order to deliver high quality patient care and the nurses’ expanding role, nursing curricula needs to be reexamined and updated to prepare future nurses to be highly competent in the present complex health care industry [23]. Many schools of nursing keep adding more knowledge and information due to the expanding growth of research. The timeline of nursing education remains the same and yet the content for students to learn is increased resulting in more student memorization, less retention of knowledge and ability to critically analyze within the clinical environment [23]. One school of nursing had implemented an active learning approach to leadership in which reflection and observation of leadership is promoted to provide a baseline for future leadership development after graduation [24]. The philosophy behind this approach is to promote leadership awareness as nurses are expected to have leadership skills within their practice. A quantitative comparative study examining transformational leadership among graduating baccalaureate nursing students (BSN) and practicing nurses showed that the BSN nursing students had significantly lower scores in transformational leadership components than the practicing nurses and the practicing nurses in leadership positions did not consider themselves better transformational leaders than the staff nurses [25]. Overall, the educational methods in preparing undergraduate and graduate nurses in academia for the present and future healthcare industry is not adequate to provide continuous success for the nurse and nurse leader [23,24,25].

In nursing, the primary focus is always the patient. At the undergraduate level of nursing studies all the skills, learning the disease entities, and being able to apply this knowledge safely at the bedside is the nucleus of nursing. Nursing work environments, turnover, retention, and interdisciplinary collaboration are not generally discussed with nursing students at a high level of understanding. Therefore, new nurses are not fully aware of the discontent within the profession of nursing. In fact, most of the time novice nurses are quite surprised that these types of issues are commonplace in the hospital work environment [2,5,9]. Many times they are confused and resort to a comfort zone of focusing on their clinical abilities and patients. Some actually leave the profession [2]. For those that stay, often they are not sure if these conditions are isolated events, temporary, or being perceived incorrectly [2,26]. Various behaviors of “settling” become the standard way of coping with these numerous circumstances of discord [26]. Eventually these methods of adjustment are not long lasting, especially if it affects patient care outcomes. Nurses, including CNOs, are all about the patient; this is their nature and to some their calling. If most of the time nurses feel they are unable to deliver the care needed for their patients, then the nurse may feel inadequate, unhappy, and unsure if they represent the caring nurse they have been taught to be [11]. The instinctual need to defend the care delivered to their patients is customary within the nursing culture.

Most of these CNOs are very good to excellent leaders despite these obstacles within their environments. This takes persistence, intelligence, communication, and courage to be successful within these circumstances. In spite of their challenges, they continued to set themselves apart from their colleagues. They were promoted without much mentoring or additional education. This additional education would have allowed them to be even more effective leaders by changing the healthcare system in a global aspect, rather than in fragments [10,12]. Nurses generally do not have a personal agenda. If allowed to be taught the true infrastructure in both the clinical and financial realms they would be the best likely change agent for healthcare. In doing so, they would broaden the inevitable process of developing beyond one’s scope.

Most of these CNOs are self-learners and highly flexible. They have had role models that were either non-nursing business executives, non-clinical nurse executives or, as most of these CNOs were, a highly clinical nurse executive. More often than not, these CNOs were being mentored by someone who may not have fully understood the nursing profession, its thinking, or processes. Knowing the whole healthcare industry is how a nurse would optimally function; it is the holistic approach and most of them are being educated in parts mixed with nursing philosophy. However, many of them believed that patient care outcomes will promote the success of the nurse, rather than first having the nurse being set up for success in order to have consistent successful patient care outcomes. How can they do this easily if the core of their profession is all about the patient and not about the nurse and the patient since they are interrelated.

## 6. Conclusions

Many of these CNOs interviewed believed they were doing the best for their nurses and healthcare facility. These interviews though, showed conflict of CNOs choosing between being an advocate for the patient or being an advocate for the nurse. Looking at their responses it was apparent that inadvertently some actually encouraged peer pressure among nurses to achieve compliance in the name of being a team player, which could make the RN feel pressured and in some cases bullied to work extra shifts. Other CNOs concluded that most nursing bedside interventions take very little time and thus the work load of an acute care RN is fairly reasonable, even though research has found many nurses believe they do not have enough time or staff to give the best care to their patients [1,2,3]. In some cases, CNOs believed that patient acuity is being addressed adequately, as critically ill patients are in the critical care areas in spite of the research stating that the workload of the RN even in Medical/Surgical units has increased due to patients being more acutely ill and their conditions more complex [1,2]. One CNO did believe that being an advocate for the nurse by making sure the nurse was set up for success would contribute greatly to the quality outcomes of patients. This CNO understood how the nurse and the patient are connected and the needs of both are required to be met in order for success in obtaining quality patient outcomes. One CNO advocated for resources for nurses in the work environment and found that not only did the nurses feel successful but quality patient outcomes were realized. As a nurse leader being an advocate for the patient involves first being an advocate for the nurse to succeed in obtaining consistent patient outcomes. A conflict between nurse productivity standards and nursing satisfaction was a concern raised by another CNO, as senior executives do not want to see unionization or poor public relations in the media concerning their facilities.

## 7. Implications

**Figure 1:**
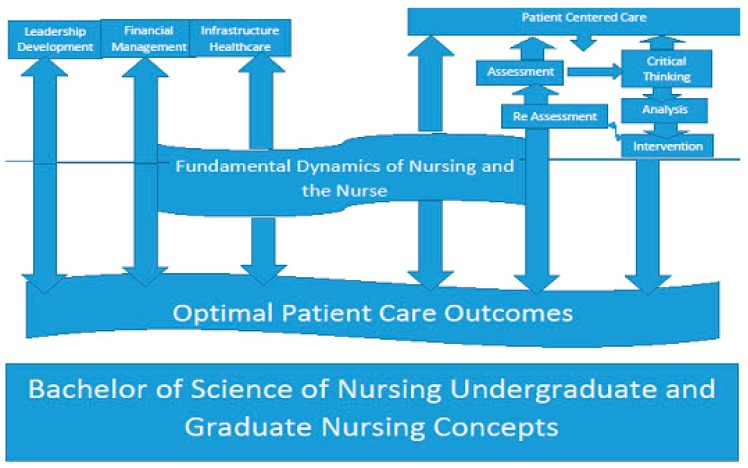
An adaption of universal Leadership and Nursing components combined by Ingwell-Spolan (2017) into the Nursing undergraduate and graduate nursing concepts.

The majority of the CNOs in these two studies experienced conflict between advocacy for the patient versus advocacy for the nurse. However, they are interrelated. Being an advocate for the nurse results in better advocacy and outcomes for the patients. Therefore, the CNO of the twenty-first century needs to have the undergraduate to graduate nursing educational foundation incorporating fundamental dynamics and relevance of the true nature of nursing, the nurse and the patient. This would include the relationship between the successful nursing environment and consistent patient care outcomes. Primary to advanced leadership development, infrastructure of the healthcare environment, financial management of healthcare systems, along with assessment, critical thinking, clinical skills, analysis and interventions of patient centered care are subjects that should be taught throughout the curriculum. The prerequisites for a BSN degree could be more inclusive of these important areas of development. The graduate level of the nursing curriculum would continue these concepts in more depth and help further hone critical thought processes for even more successful nurse executives. The results would be extremely effective for the future of nursing in all levels of leadership by performing the skills of listening, understanding, analytical reasoning, relational development, persistence, and courage within the decision making and delegating processes of nursing. The results based on the strengths and areas of potential development evidenced in these two studies across various hospital settings reveal the need to update leadership development in all levels of academia (Figure 1). This will provide the sustainability, vision, and consistency of quality patient outcomes. Knowledge is a form of power and this will promote the ability, confidence, and innovation to a profession that has had turmoil and discord for more than a century [27,28,29]. 

Change is difficult, but inevitable and necessary. Adapting to ever changing initiatives in healthcare systems, reacting to situations rather than being proactive, all lead to less than optimally effective patient care outcomes. For nursing to attain its true position in the healthcare industry, we must pursue these endeavors; moving forward with cohesive purpose, starting in academia, both undergraduate and graduate levels, threading through professional practice while respecting all nursing specialties and health care disciplines. The Nursing collaboration with academia and private sector are key for the future success of our profession’s achieving optimal patient care outcomes.
